# Simultaneous Efficient Fragmentation and Spheroidization: Cyclone Atomization Enables Defect-Free, High-Yield FeNi50 Powder

**DOI:** 10.3390/ma19132926

**Published:** 2026-07-07

**Authors:** Kai Kang, Shasha Huang, Kuanguang Hu, Qiang Han, Deliang Zhang

**Affiliations:** 1 School of Materials Science and Engineering, Northeastern University, Shenyang 110819, China; zhangdeliang@mail.neu.edu.cn; 2 School of Chemistry and Chemical Engineering, Linyi University, Linyi 276000, China; 3 Qingdao Ruinuotu Intelligent Technology Co., Ltd., Qingdao 266109, China; hhqd8985@163.com (K.H.); qiang87821@163.com (Q.H.)

**Keywords:** cyclone atomization, FeNi50 powder, atomization pressure, spherical particles, microstructure evolution

## Abstract

FeNi50 powder production for metallic magnetic cores faces challenges including low fine-powder yield and defects like hollow particles. This study employed cyclone atomization to prepare FeNi50 powder and systematically examined the effects of atomization pressure (1–6 MPa) through combined simulation, experiment, and theoretical analysis. Results show that increasing pressure reduces the average particle size (D50) from 80.7 μm to 27.9 μm and raises the fine powder yield (−500 mesh) from 19.4% to 50.0%, far exceeding that of close-coupled nozzle atomization (<10%). The powder particles are spherical/near-spherical with dense, non-hollow interiors. Higher pressure also increases the cooling rate, which blurs surface grain boundaries, refines grain structure, and induces single-crystal or amorphous characteristics in particles < 15 μm while suppressing N and O absorption. X-ray diffraction confirms the phase composition remains unchanged. These evolutions originate from three synergistic mechanisms: competition between solidification and spheroidization times, centrifugal and Magnus forces from swirling flow, and plastic-state droplet deformation imparting specific surface roughness. Cyclone atomization therefore proves a promising method for producing high-quality FeNi50 powder, suitable for large-scale manufacturing of high-performance magnetic powder cores.

## 1. Introduction

Iron–nickel soft magnetic powder, particularly FeNi50 alloy, is in high demand for miniaturized and high-frequency electronic components, such as high-frequency power inductors and integrated molded inductors, due to its high magnetic induction and excellent DC bias performance [1,2,3]. These applications strictly require the powder to possess high sphericity, a rational particle size distribution, a high yield of fine powder, and a microstructure free from internal defects (such as hollow particles) [1,2,4,5]. However, existing mainstream preparation technologies struggle to simultaneously meet these requirements. Traditional gas atomization can achieve relatively high sphericity, but the low yield of fine powder limits raw material utilization efficiency and economic viability [6,7,8,9,10]. Water atomization, while cost-effective, produces powder with poor sphericity and significantly higher oxygen content, which severely degrades soft magnetic properties, particularly for high-frequency applications [11,12,13,14]. Furthermore, both processes commonly face challenges in controlling microstructural defects such as hollow particles and satellite particles, posing a bottleneck for the industrial development of high-end soft magnetic powders [6,8,15,16].

To overcome these bottlenecks, emerging cyclone atomization technology has attracted increasing attention recently [17,18]. It generates a supersonic strongly swirling composite flow field, which synergistically couples centrifugal force, shear effect, and liquid film breakup. This flow field shears molten metal into twisted umbrella-shaped liquid films for efficient fine droplet formation. The centrifugal effect stabilizes flying droplets to reduce satellite particles, and the special flow field structure suppresses hollow particle generation by weakening direct droplet impact. Current cyclone atomization research mainly focuses on flow field simulation and theoretical exploration. Wang et al. [19] confirmed that cyclonic flow fields produce thinner liquid films, enabling finer powder with lower gas consumption. Recent studies further clarified the effects of melt superheat, atomization pressure, and nozzle structure on flow field evolution, droplet fragmentation and solidification, and constructed droplet cooling-solidification models for non-equilibrium solidification prediction [5,6,20,21]. Related work validated particle-size prediction equations and revealed the inverse correlation among particle size, cooling rate, and microstructure evolution [22,23]. Comparative analysis of mainstream atomization technologies provides industrial guidance [6], while composition-optimized iron-based soft magnetic powders with high magnetization and low core loss lay a foundation for high-performance Fe-Ni magnetic core applications [3,24].

However, research on cyclone atomization technology predominantly focuses on flow field simulation and theoretical analysis [6]. There remains a lack of systematic studies applying it to the preparation of FeNi50 powder and thoroughly investigating how process parameters (such as atomization pressure) influence powder characteristics (fine powder yield, defect control) and the underlying mechanisms. In particular, there is insufficient experimental and theoretical explanation regarding how the competitive relationship between droplet solidification time and spheroidization time determines powder morphology, and how centrifugal and Magnus forces in the swirling flow field work together to suppress the formation of satellite and hollow particles [7].

To address this, this study integrates computational fluid dynamics simulation, experimental preparation, and theoretical analysis, aiming to achieve the following objectives: (1) systematically reveal the influence of atomization pressure on the particle size distribution, fine powder yield, morphology, and internal defects of FeNi50 powder; (2) clarify the dynamic competitive mechanism of “secondary fragmentation-solidification-spheroidization” of droplets in the swirling flow field and establish the correlations between process, microstructure, and performance; and (3) explain, from the perspectives of energy transfer and time-scale competition, the advantages of cyclone atomization over close-coupled nozzle atomization [16] in achieving high fine powder yield and low-defect powder. This research will provide a theoretical basis and process window for the industrial production of high-performance FeNi50 soft magnetic powder.

Distinct from previous theoretical-oriented cyclone atomization studies, this work innovatively constructs a tripartite “process–simulation–theory” research framework and clarifies the unique application value of the coupled cyclone flow mechanism in FeNi50 powder fabrication. First, for the first time, the correlation between cyclone atomization pressure and multi-dimensional properties (particle size, morphology, defects, gas content, grain structure) of FeNi50 powder is systematically established on industrial-scale equipment. Second, the closed-loop validation of CFD simulation and experiments clarifies the synergistic effect of liquid film torsional breakup behavior under supersonic swirling flow on improving fine-powder yield. Third, a competitive model between solidification time and spheroidization time is quantified to interpret critical conditions for irregular morphology and defect formation, providing targeted theoretical guidance for industrial process regulation of FeNi50 powder.

## 2. Materials and Methods

### 2.1. Raw Materials

The FeNi50 alloy material was selected with the planned specific chemical composition, as detailed in Table 1. The iron used was pure iron (Taiyuan Iron & Steel (Group) Co., Ltd., Taiyuan, China), and nickel was obtained from Jinchuan Group Co., Ltd., Jinchang, China.

### 2.2. Powder Preparation by Cyclone Atomization

**Figure 1:**
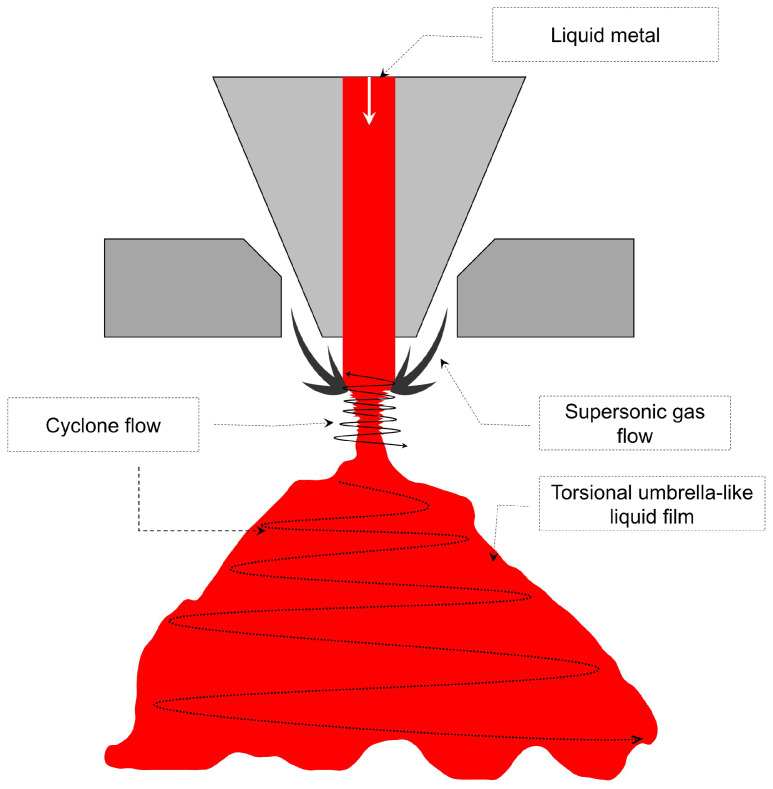
Process schematic of cyclone atomization for preparing metal powders.

Figure 1 illustrates the process principle of preparing FeNi50 alloy powder using cyclone atomization. The major role of the primary mechanism is to enhance the synergistic effect of “supersonic airflow and cyclone flow” to achieve the fragmentation of molten metal [17]. The main steps are as follows: (1) Liquid metal supply: The molten FeNi50 alloy (shown in red in Figure 1 and Video S1) flows out from the funnel structure above, forming a continuous and stable liquid column. (2) Supersonic gas flow impact: Supersonic gas flow jets are introduced from both sides of the device, delivering direct impact on the liquid column due to their high kinetic energy [25]. (3) Cyclone flow formation and liquid film transformation: The obliquely injected airflow creates a rotational flow field, which, under the combined action of “rotational shear and high-speed impact,” stretches and disperses the liquid column into a torsional umbrella-like liquid film [26]. (4) Liquid film fragmentation into powder: The umbrella-like liquid film possesses a large specific surface area and is further broken into fine droplets under continuous cyclone shear, ultimately solidifying into metal powder [27]. This process enhances atomization efficiency through the synergistic effect of “airflow kinetic energy and cyclone shear”: high-speed airflow provides the high kinetic energy required for initial dispersion, while cyclone flow enhances shear effects, promoting the transformation of the liquid column from a “columnar” to a “thin umbrella-like liquid film.” The liquid film has a larger specific surface area, creating favorable conditions for the subsequent formation of fine and uniform droplets [28].

**Figure 2:**
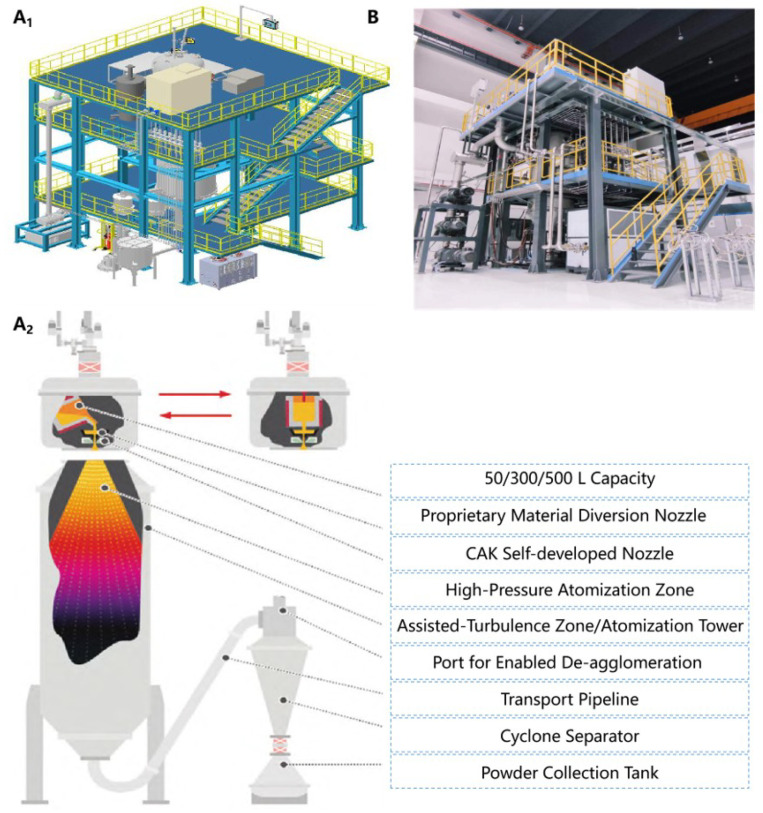
Schematic of the cyclone atomization powder preparation process: (**A_1_**) Three-dimensional layout schematic of the complete powder production equipment; (**A_2_**) Schematic illustrating the internal working mechanism and structural details of the core atomization unit. The bidirectional red arrows denote the interchangeable configuration of two types of atomization nozzles, and the color gradient inside the atomization tower reflects the temperature evolution of the molten metal stream during cooling and solidification (from high temperature in orange-yellow to low temperature in dark purple/black). (**B**) Photograph of the actual experimental device.

Figure 2 shows a simplified schematic of the cyclone atomization powder preparation process (Figure 2(A_1_,A_2_)) and an on-site photograph of the equipment (Figure 2B). The experiment used a domestically produced induction melting inert gas atomization unit, model XR-PFKG250, as the main structure, equipped with a vacuum system (vacuum level set at 101–102 Pa). Nitrogen gas (at room temperature) was used as the atomization gas during the experiments. The atomizing nozzle assembly employed a CAK-G type nozzle disc (aperture 1 mm), with the atomization pressure adjustable within the range of 1–6 MPa. A 250 kg industrial-scale crucible was used, with the molten metal temperature set at 1650 °C. A 5 mm ceramic nozzle was selected to ensure liquid stream stability. For sieving, an ultrasonic rotary sieve (model: S49-A-AC-1000-2S) was used, and ultra-fine powder classification was carried out using an airflow classifier (model: VS-V-01).

### 2.3. Structural Parameters of the Cyclone Atomizer and Atomization Process Settings

The internal structure of the cyclone gas atomization nozzle assembly adopts an insert-type delivery nozzle design, which effectively avoids flow instability and nozzle blockage risks caused by alloy liquid stream vibration. The key structural parameters of the nozzle assembly include: the extension length of the delivery nozzle (h), the airflow injection width (D), and the airflow injection angle (α). Based on the on-site conditions, the fixed structural parameters are determined as follows: airflow injection angle α = 85°, delivery nozzle extension length h = 7.6 mm, and airflow injection width D = 30 mm. The atomization process parameters are set as follows: for comparison with the actual experimental conditions, nitrogen gas is selected as the atomization gas in the simulation, with the atomization pressure adjustable in the range of 1–6 MPa and the atomization gas inlet temperature fixed at 300 K. To establish the cyclone gas flow model, the following assumptions are made: (1) the atomizing gas follows the ideal gas law, with a steady flow state that satisfies the isentropic compression law; (2) both mass transfer and momentum transfer exist between gas flow layers; and (3) the influence of gravity on the gas flow is neglected.

### 2.4. Simulation of the Cyclone Atomization

To systematically study the characteristics of the cyclone gas flow field and optimize the powder preparation process, computational fluid dynamics (CFD) technology combined with the volume-of-fluid (VOF)-to-discrete phase model (DPM) model was employed to conduct numerical simulations. This simulation method enables the characterization of the distribution characteristics, velocity, and temperature variation patterns of the gas flow field under different process parameters [29], the prediction of particle size distribution, and morphological features of the powder.

#### 2.4.1. Mathematical Model

The fluid flow follows Newton’s second law, the law of conservation of energy, and the law of conservation of mass, with fluid dynamics governed by the momentum equation, energy equation, and continuity equation, respectively [30]. A three-dimensional cylindrical model with a diameter of 60 mm and a height of 100 mm (including the delivery nozzle and annular cavity) was constructed. To save storage space and computational time, one-quarter of the model was adopted for calculation, with symmetry planes defined on the section faces. Mesh generation was performed using Ansys meshing, with 1,383,219 nodes and 4,071,488 elements. A smooth transition method was applied (transition ratio: 0.272), with a maximum of 10 boundary layers, a growth rate of 1.2, and a minimum mesh size of 0.1 mm. The inlet boundary condition of the cyclone atomizer is defined as a pressure inlet, with pressure ranging from 1 to 6 MPa (referenced to absolute pressure), and the inlet temperature of the atomizing gas ($N_{2}$) fixed at 300 K. The outlet boundary condition of the atomization chamber is set as a pressure far-field boundary, and all walls are configured as adiabatic no-slip walls. Pressure variation inside the atomization chamber is negligible compared with the inlet total pressure. For nitrogen at 300 K within 1–6 MPa, the deviation of the compressibility factor is less than 5%, which validates the ideal gas assumption and achieves a trade-off between computational efficiency and calculation accuracy. The inlet total pressure of the nozzle plate is strictly set according to experimental parameters. The far-field outlet boundary condition is adopted because the volume of the atomization chamber is far larger than the jet expansion zone, which avoids back-reflection of pressure perturbations. The physical parameters of the atomizing gas ($N_{2}$) are set as follows: density $1.25\;{\mathrm{kg}\cdot m}^{-3}$ (ideal gas state), thermal conductivity $0.0242\;{W\cdot m}^{-1}\cdot K^{-1}$, specific heat capacity $1.038\;{J\cdot \mathrm{kg}}^{-1}\cdot K^{-1}$, molar mass $28.0134\;{g\cdot \mathrm{mol}}^{-1}$. The gas viscosity follows the Sutherland formula, with the expression [31]:

(1) $$\mu =\mu_{0}{\left(\frac{T_{g}}{T_{0}}\right)}^{\frac{3}{2}}\frac{T_{0}+S}{T+S}$$

where μ is the gas viscosity; $\mu_{0}$ is the gas viscosity under standard conditions, taken as $1.663\times 10^{-5}\;{\mathrm{kg}\cdot m}^{-1}\cdot s^{-1}$; $T_{0}$ is the standard temperature; $T_{g}$ is the thermodynamic temperature of the gas; and *S* is the Sutherland constant. The coupled algorithm was selected for implicit solving, with a second-order scheme adopted for the turbulence model and the Courant number set to 2. When the computational residuals fell below $10^{-7}$, the calculation was judged as converged, and iterations were terminated. For multiphase flow simulation, the VOF model was employed, and an optimized DPM model was chosen for the fragmentation process. An adaptive mesh model was applied at the gas–liquid interface to enhance mesh resolution and computational accuracy [31].

#### 2.4.2. Grid Independence Analysis and Boundary Conditions

To ensure the grid independence of the simulation results, three sets of tetrahedral/mixed grids with varying densities were constructed: coarse (∼2 million elements), medium (∼4 million elements, as used in the main text), and fine (∼6 million elements). Comparing the gas velocity distribution on key monitoring planes (Z=0, 50, 100 mm) and the calculated Sauter mean diameter (SMD) of droplets, the difference between the medium and fine grid results was found to be less than 2%. Therefore, using the medium grid with approximately 4 million elements ensures accuracy while significantly improving computational efficiency. The boundary conditions were set as described in the main text. It is worth emphasizing that the outlet was configured as a pressure far-field boundary to better simulate the open flow within the atomization chamber. The walls were treated with no-slip, adiabatic conditions, and the standard wall function was enabled.

#### 2.4.3. Model Limitations

Although the current CFD model successfully reproduces the variation trend of powder particle size with atomization pressure, four inherent limitations remain: (1) Two-dimensional axisymmetric simplification: Despite local mesh refinement, the 2D model simplifies the three-dimensional swirling flow into axisymmetric flow, which cannot fully capture asymmetric oscillation of the vortex core and circumferential non-uniformity. (2) Ideal gas and isentropic flow assumptions: The model treats atomization gas as an ideal gas with isentropic flow, while non-equilibrium condensation and subsequent physical property deviation during high-pressure gas expansion at the nozzle exit are neglected. (3) Neglected inter-droplet interaction: The DPM model excludes droplet collision--coalescence behaviors, which may underestimate the fraction of ultrafine powder and induce deviation in predicted particle-size distribution width. (4) Simplified thermal boundary condition: The chamber wall is set as adiabatic, whereas the actual wall temperature rises continuously during atomization and alters the internal gas temperature field.

#### 2.4.4. Dimensionless Scaling and Relative Importance of Forces Acting on Droplets

To quantitatively evaluate the hydrodynamic forces on droplets within the swirling flow field, dimensionless analysis was carried out under representative cyclone gas atomization conditions (atomization pressure: 3–5 MPa, droplet diameter: 5–150 μm). Global flow-field dimensionless numbers including Reynolds number (Re), Weber number (We), Bond number (Bo), and Ohnesorge number (Oh) were calculated preliminarily. For metal droplets smaller than 200 μm with high flight velocity, We≫Bo, confirming gravity can be reasonably neglected; moderate We further indicates that surface tension dominates droplet spheroidization behavior. Detailed dimensionless scaling analysis of individual droplet forces is elaborated as follows:

(1) Centrifugal force

Droplets rotate rapidly with the swirling gas flow, with centrifugal acceleration defined as $\alpha_{c}=u_{\theta }^{2}/r$, where $u_{\theta }$ is the tangential velocity of atomized droplets and *r* represents radial position. The centrifugal Froude number is proposed as:

(2) $$\mathrm{Fr}=\frac{\rho_{l}u_{\theta }^{2}/r}{\rho_{g}u_{r}^{2}/L_{c}}\approx \frac{\rho_{l}}{\rho_{g}}\cdot \frac{u_{\theta }^{2}}{u_{r}^{2}}\cdot \frac{L_{c}}{r}$$

according to numerical simulation results, the tangential velocity $u_{\theta }=200$–400 m/s, radial velocity $u_{r}=50$–100 m/s, and the characteristic length $L_{c}$ is approximately equal to radial position *r*. The calculated Fr=1–5, demonstrating that centrifugal force is comparable to inertial force, which acts as the core driving force for droplet spatial separation and liquid film spreading.

(2) Magnus force

High-speed rotation of droplets in the swirling flow field generates Magnus lift force, with the expression $F_{M}=C_{M}\cdot \rho_{f}\cdot \frac{\pi d^{3}}{8}\cdot \omega v_{p}$, where ω denotes droplet angular velocity. The ratio of Magnus force to inertial force is defined as:

(3) $$\Pi_{M}=\frac{|F_{M}|}{m_{p}a_{p}}\approx \frac{3}{4}\cdot \frac{\rho_{g}}{\rho_{l}}\cdot \frac{\omega D}{v_{p}}$$

based on typical shear rates of supersonic gas flow, the spin frequency of droplets ranges from $10^{4}$ to $10^{5}$ s^−1^. Taking $\omega D/v_{p}=0.1$–0.5, we obtain $\Pi_{M}=0.2$–0.8. Therefore, Magnus force cannot be neglected for medium- and large-sized droplets, as it significantly affects their lateral migration and morphological evolution.

(3) Thermophoretic force

Thermophoretic force is induced by temperature gradients, described as $F_{\mathrm{th}}=-\frac{6\pi \mu_{g}^{2}D}{\rho_{g}T}\cdot \frac{k_{g}}{2k_{g}+k_{p}}\nabla T$. The ratio of thermophoretic force to inertial force is defined as:

(4) $$\Phi_{\mathrm{th}}=\frac{|F_{\mathrm{th}}|}{m_{p}a_{p}}\approx \frac{18\mu_{g}^{2}}{\rho_{p}\rho_{g}D^{2}a_{p}T}\cdot \frac{k_{g}}{2k_{g}+k_{p}}\left|\nabla T\right|$$

taking the temperature gradient in the atomization zone $\left|\nabla T\right|=10^{6}$ K/m, $\Phi_{\mathrm{th}}=0.1$–0.3 for droplets with *D* < 10 *μ*m, while $\Phi_{\mathrm{th}}<0.02$ for *D* > 30 *μ*m. Thus, thermophoretic force mainly influences the motion trajectory of ultrafine droplets and contributes to the left shift of the overall particle-size distribution peak.

(4) Other minor forces

The particle Reynolds number is defined as Rep=ρgDvp/μg, ranging from 1 to 500 under current atomization conditions, indicating a transitional flow regime. The ratio of Basset force to inertial force is generally less than 0.05 when Rep>10, which can be reasonably neglected.

The above dimensionless parameters are summarized in Table 2, quantitatively supporting the inclusion of Magnus force and thermophoretic force in the physical model, as well as the rationality of neglecting Basset force and other minor forces. Combined with the solidification–spheroidization competition analysis in subsequent sections, the multi-mechanism regulation of cyclone-atomized powder morphology and particle-size distribution is fully validated.

### 2.5. Powder Characterization

The chemical composition of the FeNi50 alloy powder was determined by inductively coupled plasma optical emission spectrometry (ICP-OES, Agilent 720ES, USA). For sample preparation, 0.1 g of alloy powder was dissolved in a mixed acid solution (HCl:HNO_3_ = 3:1, *v*/*v*) under heating at 80 °C. The resulting solution was diluted to 100 mL with deionized water. The concentrations of Fe and Ni were measured at characteristic wavelengths of 259.940 nm and 231.604 nm, respectively, using standard solutions for calibration. All measurements were performed in triplicate, and the average mass percentages are reported in Table 1.

After the atomization process was completed and the temperature dropped to room temperature, the collection chamber was opened, and the fully cooled powder collected. All atomization experiments under each pressure (1–5 MPa) were independently repeated three times to ensure reproducibility.

Laser particle size analysis: Put the prepared FeNi50 powder to water, fully disperse agglomerated particles via ultrasonic vibration, and ensure uniform distribution of particles throughout the system using an electromagnetic circulating water pump. Then, use the Malvern Mastersizer 3000E laser particle size analyzer to measure the particle size distribution, with a measurement range of 0.1–1000 μm. Each sample was tested three times, and the average value was taken as the final result.

X-ray diffraction (XRD) analysis: Sieve the FeNi50 powder obtained under different atomization pressures through a 60-mesh sieve. Use a PANalytica Empyrean XRD instrument with Cu $K_{\alpha }$ radiation (λ = 1.54056 Å) for testing. The scanning range is 5–90° at a speed of 0.5°/min. XRD tests were repeated twice for each sample to verify the stability of phase results.

To observe the surface and cross-sectional morphology of the FeNi50 powder, first perform deoxygenation and dehumidification treatment on the powder; then, [p] fix it with conductive adhesive to prepare samples for scanning electron microscopy (SEM). Backscattered electron imaging can be used to observe the particle morphology and surface features. For cross-sectional analysis, the powder is fixed on a copper plate by nickel electroplating, approximately half of the coating is removed by grinding, and the surface is polished smooth with 5000-grit sandpaper to observe the internal microstructure. A Hitachi SU8010 SEM is used, operated at an acceleration voltage of 3 kV under high-vacuum conditions, supplemented by optical metallographic microscopy for observation. To observe the surface and cross-sectional morphology of the FeNi50 powder, first perform deoxygenation and dehumidification treatment on the powder; then, fix it with conductive adhesive to prepare samples for scanning electron microscopy (SEM). Backscattered electron imaging can be used to observe the particle morphology and surface features. For cross-sectional analysis, the powder is fixed on a copper plate by nickel electroplating, approximately half of the coating is removed by grinding, and the surface is polished smooth with 5000-grit sandpaper to observe the internal microstructure. A Hitachi SU8010 SEM is used, operated at an acceleration voltage of 3 kV under high-vacuum conditions, supplemented by optical metallographic microscopy for observation.

Metallographic samples were prepared using a QATM Saphir 560 polishing machine: grinding was performed sequentially with 240-, 1000-, and 2000-grit SiC sandpapers for rough grinding, followed by fine grinding with 5000- and 8000-grit sandpapers (30 s per step). Polishing was then carried out with 2.5 μm diamond suspension on a nylon cloth (300 rpm, 20 N load, 3 min) and 1 μm diamond suspension on a velvet cloth (500 rpm, 10 N load, 3 min). The polished samples were etched with dilute aqua regia at 25 °C for 30 s, and metallographic observations were performed using an Olympus BX-53 optical microscope.

## 3. Results and Discussion

### 3.1. Simulation Results

The TAB breakup model is selected based on the energy transfer characteristics of cyclone atomization, with key assumptions justified as follows: (1) Modified applicability of the TAB breakup model: Droplet breakup in cyclone atomization is driven by shear and centrifugal stretching from high-speed swirling flow, analogous to forced vibration in a spring-mass system. The TAB model describes this process via droplet vibration-deformation-breakup based on the Weber number. A correction factor correlated with swirling shear rate is introduced to approximate cyclone effects, achieving a practical trade-off between computational efficiency and physical description. (2) Neglected liquid film primary breakup stage: The VOF-to-DPM conversion assumes instantaneous transformation of the liquid film into spherical droplets at a critical position, without resolving full liquid film fluctuation and perforation processes. This assumption is reasonable because the umbrella-shaped liquid film in cyclone atomization is extremely thin, and its primary breakup duration is far shorter than droplet flight time, exerting limited influence on the final particle-size distribution. (3) Simplification of secondary forces: Magnus force and thermophoretic force are incorporated to describe lift effects of swirling flow and particle migration induced by temperature gradients. Basset force and Saffman force are neglected, as their time scales and magnitudes are 1–2 orders of magnitude lower than dominant forces under the working conditions of this study.

**Figure 3:**
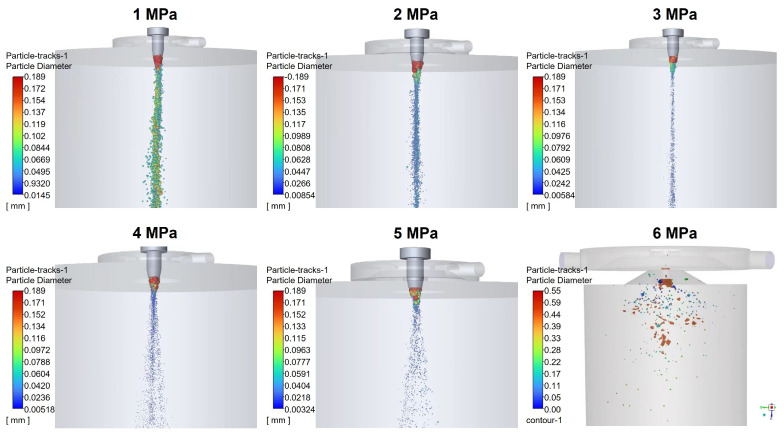
Gas–liquid two-phase simulation diagrams at atomization pressures of 1–6. MPa During the gas–liquid two-phase flow simulations, there were multiple computational failures.

Figure 3 displays three-dimensional numerical simulation diagrams of the gas–liquid two-phase flow under different atomization pressures, clearly illustrating the process of molten metal fragmentation into fine metal droplets under varying conditions. At a pressure of 1 MPa, the iron–nickel alloy stream is incompletely fragmented, resulting in low atomization efficiency. The coarsely fragmented powder particles are primarily concentrated along the negative *Z*-axis direction, with an average particle size of 80–90 μm. At 2 MPa, atomization efficiency improves significantly, with increased fragmentation of the alloy stream. The diameter of powder particles distributed along the *Z*-axis decreases, yielding an average particle size of 40–50 μm. At 3 MPa, atomization efficiency further improves compared to 2 MPa, with an increased proportion of fine powder particles and an average particle size of 35–45 μm. When the pressure is raised to 4 MPa, atomization performance becomes favorable. A hollow cylindrical region along the *Z*-axis begins to emerge, with metal droplets formed from the fragmented alloy stream distributed symmetrically along the *Z*-axis. The powder particles are further fragmented by the cyclonic effect and distributed on both sides of the center, with the average particle size further decreasing to 30–35 μm. At 5 MPa, the atomized droplets formed from the fragmented alloy stream exhibit a “funnel-shaped” distribution within the atomization chamber. Fine atomized droplets below the fragmentation point spread symmetrically along the *Z*-axis [8]. Compared to pressures of 2–4 MPa, this axisymmetric distribution feature is more pronounced, the distribution area is wider, the droplets experience more uniform forces, and the particles are finer, with an average particle size of 20–30 μm.

Due to the extreme high-temperature, high-velocity, and closed conditions of supersonic atomization, direct velocity field measurement is infeasible. Accordingly, two self-consistent verifications are adopted to validate the flow-field reliability. First, the maximum relative deviation of steady-state mass flow rate between inlet and outlet boundaries is less than 0.5%, guaranteeing numerical convergence and mass conservation. Second, the simulated expansion and compression wave structures at the nozzle exit, including incident shock waves and Mach disks, qualitatively match the schlieren results of similar annular-swirl nozzles reported in the literature, confirming the accuracy of the predicted flow-field wave structures.

Simulation mechanism analysis shows that extracting data from the simulated flow field reveals that as pressure increases, the airflow velocity downstream of the nozzle outlet increases significantly, and the area of high-speed swirling flow expands. Grid independence verification and three repeated calculations were carried out to ensure the reproducibility and reliability of the simulation results. This results in a substantial enhancement of shear stress acting on the metal liquid column and initial liquid film, which is the fundamental reason for achieving more thorough primary and secondary fragmentation. Additionally, the stronger and more stable vortex structure formed under high pressure generates a significant outward centrifugal stabilizing effect on the droplets, helping to reduce droplet collisions. This mechanism explains the observed reduction in satellite particles in experiments.

Further indirect validation of droplet behaviors is provided in this study. Although direct high-speed imaging of individual droplets is unavailable, the simulated droplet characteristics agree well with macroscopic experimental and engineering phenomena. At 5 MPa, the simulated funnel-shaped symmetric droplet dispersion corresponds to uniform powder morphology and low satellite particle content in experiments, as droplet spatial distribution dominates inter-droplet collision behavior. In addition, the numerical divergence caused by recirculation zone expansion above 6 MPa is highly consistent with the back-blow clogging failure observed in practical atomization processes, further demonstrating the physical reliability of the proposed CFD model.

During the gas–liquid two-phase flow simulations, multiple computational failures occurred. After several model replacements, the DPM was ultimately adopted. In the comprehensive force analysis, additional forces such as the Magnus force and thermophoretic force were included. Traditional fragmentation models (e.g., pure aerodynamic fragmentation models) do not account for the rotational shear effects of swirling flow fields, which do not align with the “rotation-shear synergistic fragmentation” mode of cyclone atomization. Therefore, these models cannot accurately describe the fragmentation process of this technology [32,33]. Furthermore, during the gas–liquid two-phase simulations, it was found that when the airflow pressure exceeds 6 MPa (Figure 3), issues such as blockage, non-convergence of calculations, and termination of runs occur. This closely aligns with engineering practice: in actual atomization processes, pressures exceeding 5 MPa are prone to cause backflow and nozzle blockage. The core reason is that excessive pressure intensifies the impact of airflow at the outlet of the fragmentation zone, increasing the reverse backflow force above the gas–liquid interaction point. This causes the airflow to push upward, ultimately leading to atomization failure. The computational failure (backflow, blockage) observed in simulations at 6 MPa aligns with the mechanism of atomization failure in actual engineering due to excessive reverse flow force above the airflow impact point, confirming the model’s ability to capture key physical phenomena. Therefore, in practical cyclone atomization experiments, the selected atomization pressure range is 1–5 MPa.

### 3.2. Influence of Atomization Pressures on Powder Characteristics

#### 3.2.1. Model Validation

**Figure 4:**
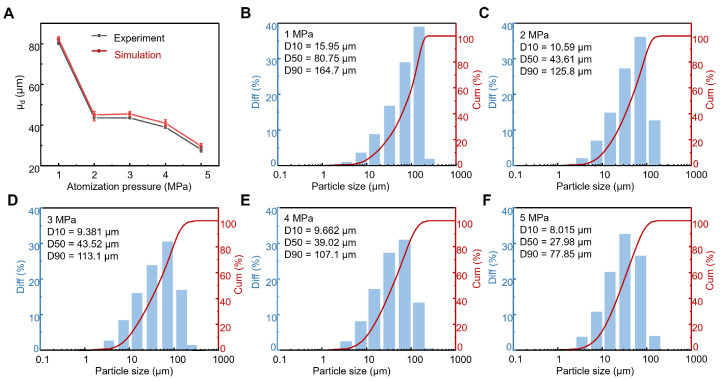
(**A**) Comparison of experimental and simulated average particle sizes of powder under different pressures. (**B**–**F**) Particle size distribution of experimentally obtained powder under different pressures (1–5 MPa).

To validate the reliability of the VOF-to-DPM coupled model established in this study, a two-step verification was conducted: (1) Flow field verification: The simulated static pressure distribution along the central axis downstream of the nozzle without melt injection was compared with the theoretically predicted Mach disk position based on classical supersonic underexpanded jet theory. The results showed good agreement. (2) Result verification: Figure 4A compares the variation in average particle size ($\mu_{d}$) with pressure obtained from experiments and simulations. As shown in the figure, the trends of both are highly consistent, with good fitting results. Combined with the mean particle size ($\mu_{d}$), variance ($\sigma_{d}$), and error parameters from Table S1, it is evident that as atomization pressure increases, $\mu_{d}$ continuously decreases, indicating that the powder refinement rate gradually improves with rising pressure. Additionally, the simultaneous reduction in $\sigma_{d}$ further validates the conclusion that particle size distribution becomes more concentrated under high pressure. The relative errors between experimental and simulated values are all controlled within 10%, falling within a reasonably acceptable range.

#### 3.2.2. Influence of Atomization Pressures on Powder Size

Figure 4B–E present the actual experimental particle size distribution results of iron–nickel alloy powder prepared by cyclone atomization. It can be observed that under an atomization pressure of 1 MPa, D10 is 15.95 μm, D50 is 80.75 μm, and D90 is 164.7 μm. As the pressure increases, under a pressure of 5 MPa, D10 becomes 8.015 μm, D50 becomes 27.98 μm, and D90 becomes 77.85 μm. All data are presented as mean ± standard deviation (SD) of three independent experiments (Figure 4A). Additionally, the yield of fine powder increases with rising pressure, and the overall peak shifts to the left. It is evident that at 1 MPa, atomization is incomplete, efficiency is low, and the interaction between the airflow and the alloy liquid stream is poor, resulting in a higher proportion of larger particles. D50 is 80.75 μm, indicating relatively coarse powder. Under atomization pressures of 2 MPa and 3 MPa, D50 values are 43.61 μm and 43.52 μm, respectively, showing similar particle sizes. However, the peak at 2 MPa is higher, and the average particle size is more concentrated compared to 3 MPa, with an increased proportion of median particle sizes. This is likely because increasing the pressure from 1 MPa to 2 MPa creates a relatively stable low-pressure zone, leading to the formation of a stable low-pressure cyclone atomization structure during secondary fragmentation. In engineering practice, a stable pressure of 2 MPa is often used to produce powder with an average particle size of 45–50 μm for applications such as spraying or laser cladding. When the pressure is increased from 4 MPa to 5 MPa, the fragmentation of the alloy liquid stream by the high-pressure airflow is further enhanced, and the kinetic energy increases, reducing the average particle size from 39.02 μm to 27.98 μm.

**Figure 5:**
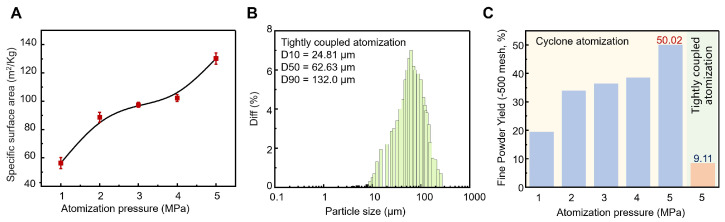
(**A**) The curve of powder specific surface area versus atomization pressure. (**B**) Particle size distribution diagram of −60 mesh powder obtained by close-coupled nozzle atomization (3 MPa). (**C**) Comparison of the −500 mesh fine powder yield for two atomization processes under different pressures (cyclone atomization: 1–5 MPa, close-coupled nozzle atomization: 5 MPa).

Figure 5A presents the trend of the specific surface area of FeNi50 powder with varying pressures. As the pressure increases, the specific surface area rises from 50.3 m^2^/kg to 130.2 m^2^/kg, showing an overall upward trend. Since the specific surface area of the powder is negatively correlated with particle size, this trend aligns with and corroborates the earlier conclusion of “increased pressure leading to finer powder”. From the perspective of atomization mechanisms, Lubanska proposed the widely used powder particle size equation, as follows [7]:

(5) $$\frac{D_{50}}{D}=K\sqrt{\frac{v_{L}}{v_{g}W_{e}}}\left(1+\frac{M}{A}\right)$$

(6) $$W_{e}=\frac{\rho_{L}V^{2}D}{\gamma }$$

where *D* is the inner diameter of the delivery nozzle (liquid stream diameter); *K* is a constant parameter depending on atomization conditions, with values ranging from 40 to 50; $v_{L}$ (m^2^/s) and $v_{g}$ (m^2^/s) are the kinematic viscosities of the molten liquid and atomizing gas, respectively; and $W_{e}$ is the Weber number. *M* (kg/s) and *A* (kg/s) are the mass flow rates of the melt and gas, respectively; $\rho_{L}$ and γ are the density and surface tension of the molten metal, respectively; and *V* is the velocity of the gas impacting the melt. In the mechanism of secondary fragmentation in cyclone atomization, the relationship between the Weber number and the degree of fragmentation is analyzed. Under the action of the cyclone airflow, gas impact on the powder exhibits multidimensional characteristics. Due to the combined effects of centrifugal force and other factors, as the atomization pressure increases, fragmentation efficiency improves, atomization efficiency rises, and the Weber number increases, leading to a significant reduction trend in the average particle size.

According to the law of conservation of energy, in the gas atomization process of metal powders, gas kinetic energy serves as the primary driving force. During atomization, kinetic energy gradually converts into surface energy. Therefore, the classical average particle size formula is referenced, as shown in the following equation [34]:

(7) $$D_{50}=C\frac{G_{m}}{G_{g}}\times \frac{\sigma_{m}}{\rho_{m}\cdot V_{g}^{2}}$$

where $D_{50}$ is the average particle size of the powder; $G_{m}$ and $\sigma_{m}$ are the mass flow rate and surface tension of the molten metal, respectively; *C* is a constant; $G_{g}$ is the mass flow rate of the gas; $\rho_{m}$ is the density of the molten metal; and $v_{g}$ is the velocity of the atomizing gas flow. Therefore, by integrating with the previous equation, it can be seen that as the pressure increases, the $v_{g}$ rises, the Weber number increases, and the average particle size decreases. $D_{50}$ is inversely proportional to the square of $v_{g}$, which also validates the conclusions of earlier research from the perspective of conservation laws.

The $v_{g}$ caused by pressure is the most direct factor influencing powder particle size. Of course, factors such as melting temperature, melt characteristics, type of atomizing gas, delivery nozzle diameter, and nozzle disc type also affect the particle size distribution of the powder [25]. These will not be analyzed one by one in this discussion. However, it is worth mentioning that there are significant differences in practical engineering applications between cyclone atomization and the currently used close-coupled nozzle atomization process, particularly in terms of fine powder yield. As shown in Figure 5B, which displays the particle size distribution of nickel-based alloy powder obtained using the close-coupled nozzle atomization process under a 3 MPa atomization pressure, it can be observed that under the same 3 MPa atomization pressure, the $D_{50}$ for the cyclone atomization process is 43.52 μm, while the average particle size for the close-coupled nozzle atomization process is 62.6 μm, indicating a considerable difference.

For powder manufacturing enterprises, the yield of ultrafine powder (−500 mesh) is a core competitive indicator that determines the applicability of powders for downstream high-end materials. As shown in Figure 5C and Tables S2–S6, the ultrafine powder yield of cyclone atomization rises from 19.41% to 50.02% with increasing pressure, far exceeding the yield (<10%) of conventional close-coupled atomization. This superiority originates from distinct airflow field structures: close-coupled atomization depends on concentrated impact near the convergence point [35], whereas cyclone atomization utilizes the synergistic effect of supersonic and swirling flow to form a high-speed rotating umbrella-shaped gas curtain, which continuously shears and stretches the molten metal column with higher energy transfer efficiency and more sufficient fragmentation. As further validated by Table 3, compared with mainstream gas, water, and centrifugal atomization, cyclone atomization achieves higher fine powder yield under moderate pressure, along with extremely low hollow particle fraction, negligible satellite particles, low oxygen content, and elevated apparent density. These comprehensive advantages demonstrate its great potential for fabricating high-quality FeNi50 soft magnetic fine powders for high-frequency applications.

#### 3.2.3. Influence of Atomization Pressures on Powder Morphology and Microstructure

**Figure 6:**
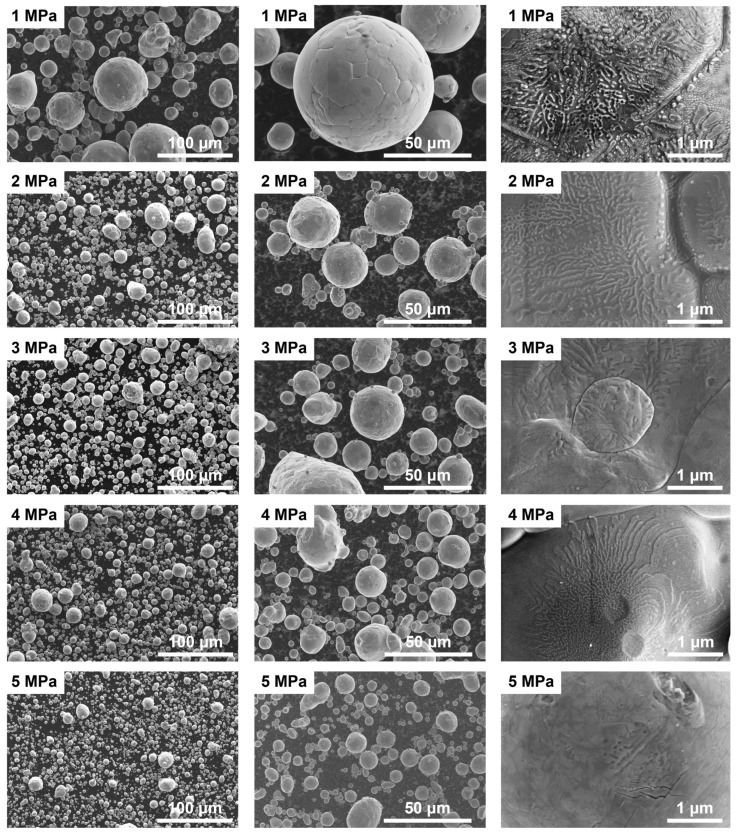
SEM images of −60 mesh FeNi50 powder prepared under different atomization pressures.

Figure 6 shows SEM images of −60 mesh FeNi50 powder prepared under different pressures. The powder displays a spherical or near-spherical morphology under all conditions. Even at 1 MPa, no obvious flake-like particles are observed, indicating that the cyclone atomization process achieves effective fragmentation and spheroidization even at low pressure. Further analysis of particle size, distribution uniformity, and surface morphology reveals the following trends: (1) Particle size decreases significantly with increasing pressure. At 1 MPa, the powder contains many coarse particles (some ≥ 100 μm) along with a few fine ones. As pressure rises to 5 MPa, coarse particles largely disappear, and the powder mainly consists of fine particles tens of micrometers in size. This agrees with the “secondary fragmentation” mechanism in gas atomization: higher gas pressure provides greater kinetic energy, leading to more thorough droplet breakup and smaller final particle size. SEM-based statistics (Figure S1 and Table S7) show that the proportion of large particles (>50 μm) decreases rapidly and fine particles (10–40 μm) increase significantly with rising atomization pressure, consistent with the declining D50 trend (80.75 → 27.98 μm) from laser diffraction (Figure 4). The slightly larger laser-measured D50 results from minor particle agglomeration, while SEM statistics reflect true single-particle sizes, cross-verifying the reproducibility and reliability of particle size data. Further analysis confirms the powder exhibits a unimodal particle size distribution, and the broad distribution tail at low pressure results in a wide size range. Triplicate independent experiments were conducted for each pressure, with all quantitative data expressed as mean ± standard deviation. (2) Particle size distribution becomes more uniform at higher pressure. At 1 MPa, particle sizes vary widely, with a mix of coarse and fine particles. From 2 to 5 MPa, the size distribution becomes more concentrated, with the best uniformity achieved at 5 MPa. This is due to enhanced secondary fragmentation under high pressure, which thoroughly breaks droplets of different sizes, yielding a more consistent powder. (3) Surface morphology evolves obviously with increasing pressure. Higher atomization pressure reduces particle size and increases fine powder fraction. The elevated cooling rate gradually blurs surface grain boundaries, and distinct grain boundaries are hardly observed at 5 MPa.

**Figure 7:**
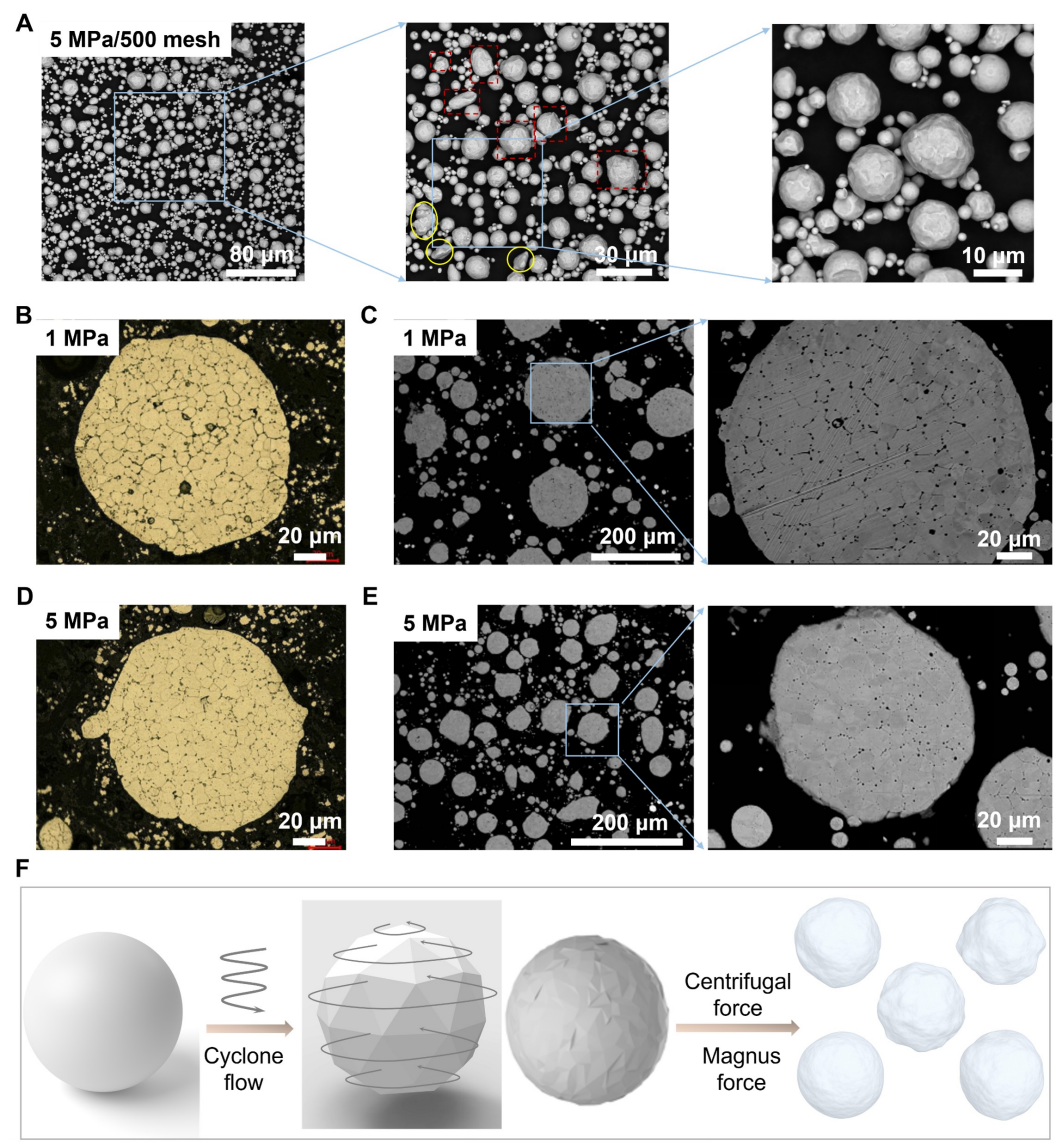
(**A**) SEM images of −500 mesh FeNi50 powder prepared under 5 MPa. In this panel, blue solid squares indicate the selected regions for stepwise high-magnification observation; red dashed squares mark the irregular non-spherical powder particles; yellow circles denote the fine satellite particles adhered to the coarse powder surface. Internal microstructure of powder under different pressures: (**B**,**D**) optical metallographs and (**C**,**E**) SEM images (pressures: 1 MPa and 5 MPa). (**F**) Schematic diagram of the process for preparing rough spherical powder via cyclone atomization.

Additionally, as the pressure increases, the likelihood of forming special powder types (primarily including irregular particles, hollow particles, and satellite particles) rises. Further observation of the −500 mesh fine powder under 5 MPa (Figure 7A) identifies several typical morphologies: within the red dashed box, there are near-spherical particles with rough surfaces, resembling “potato-like” shapes; a small number of “dumbbell-shaped” particles are also visible. Irregular particles with non-sharp edges are marked by yellow circles. Notably, no satellite particles were found in Figure 7A, and only a small amount of soft agglomeration of fine powder was present, which can be dispersed in subsequent processing steps.

Typically, the probability of hollow particle formation increases when molten metal droplets are exposed to direct airflow or when the airflow weakens. Therefore, in the design of the cyclone atomization nozzle, rotational vortex flow acting on the metal droplets is considered as an alternative to direct airflow to reduce internal defects. To verify the effectiveness of this design, the internal structures of powder produced under 1 MPa and 5 MPa atomization pressures were observed using an optical microscope (Figure 7B,D) and SEM (Figure 7C,E), respectively. The results show that as the pressure increases from 1 MPa to 5 MPa, the internal structure and grain size of the FeNi50 powder undergo significant changes. Notably, under all pressure conditions, no hollow structures were observed in the powder [36].

Regarding internal defects, optical microscope and SEM images under 1 MPa conditions (Figure 7B,C) reveal the presence of numerous fine pores and dispersed cavities in the powder. This is due to the relatively weak wrapping and constraining effect of the rotational vortex flow on droplets under low pressure. Some droplets remain exposed to “direct airflow” or insufficient airflow coverage, preventing internal gases from being discharged in a timely manner during solidification and resulting in solid structures containing pores. When the pressure increases to 5 MPa (Figure 7D,E), the number of internal pores in the powder significantly decreases, the size of cavities is reduced, and the overall structure becomes denser. This indicates that the enhanced swirling effect effectively promotes gas discharge, further improving the compactness of the powder. Regarding grain size, a comparison of SEM images (Figure 7C,E) shows that the powder grains under 1 MPa are coarser with clearer grain boundaries, while those at 5 MPa exhibit much finer grain structures. At low pressure of 1 MPa, larger droplets cool slowly with a more uniform solidification environment, allowing sufficient time for grain growth and resulting in relatively coarse grains. By contrast, the higher atomization pressure at 5 MPa produces smaller droplets with a greatly elevated cooling rate, which strongly inhibits grain growth and refines the microstructure. Rapid solidification even induces single-crystal or amorphous features in fine particles below 15 μm, further blurring surface grain boundaries.

Furthermore, although cyclone atomization significantly reduces the formation of hollow and satellite particles, the resulting powder is not perfectly spherical but exhibits rough, near-spherical morphological characteristics due to the action of high-speed rotating airflow on the metal droplets. The formation process is illustrated in Figure 7F. During the secondary fragmentation stage, molten metal droplets are influenced by the cyclone airflow, initiating a transformation in surface morphology: under the action of thermophoretic forces, the outer layer predominantly adheres to fine powder with smaller sizes (<20 μm), while the inner layer consists mainly of particles with larger sizes (>30 μm). The inner particles, subjected to higher airflow velocities, experience enhanced tangential forces, leading to the formation of polyhedral structures as their surfaces are cut by the cyclone flow. Subsequently, under the combined effects of the Magnus force and centrifugal force, these particles gradually evolve into near-spherical or surface-rough powder. As the particle size increases (>30 μm), the probability of forming rough near-spherical powder also rises, a phenomenon already validated in the earlier SEM images.

#### 3.2.4. Powder Composition and Phase Analysis

**Figure 8:**
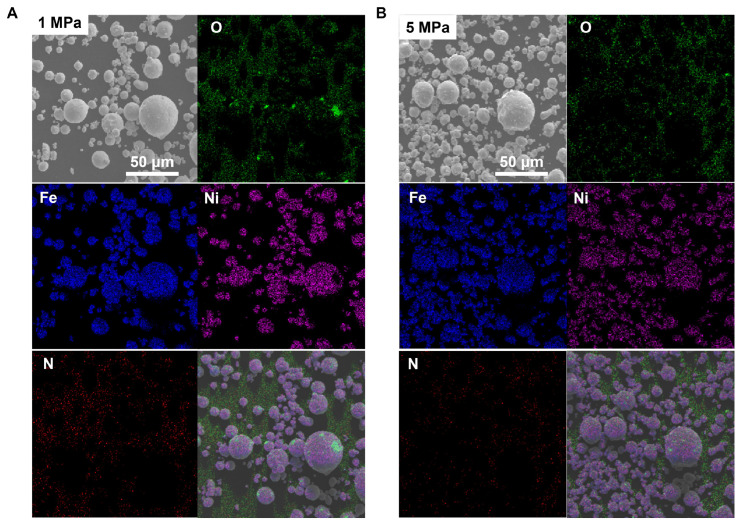
Energy-dispersive spectroscopy analysis of powders under (**A**) 1 MPa and (**B**) 5 MPa pressures.

FeNi50 powders produced under different pressures are composed primarily of two main elements, Fe and Ni (Figure 8). Due to atomization being conducted in a nitrogen atmosphere and air environment, trace amounts of N and O elements are detected in the spectra. It can be observed that as the pressure increases from 1 MPa to 5 MPa, the cooling rate rises, leading to a reduction in the powder’s absorption of gaseous elements.

**Figure 9:**
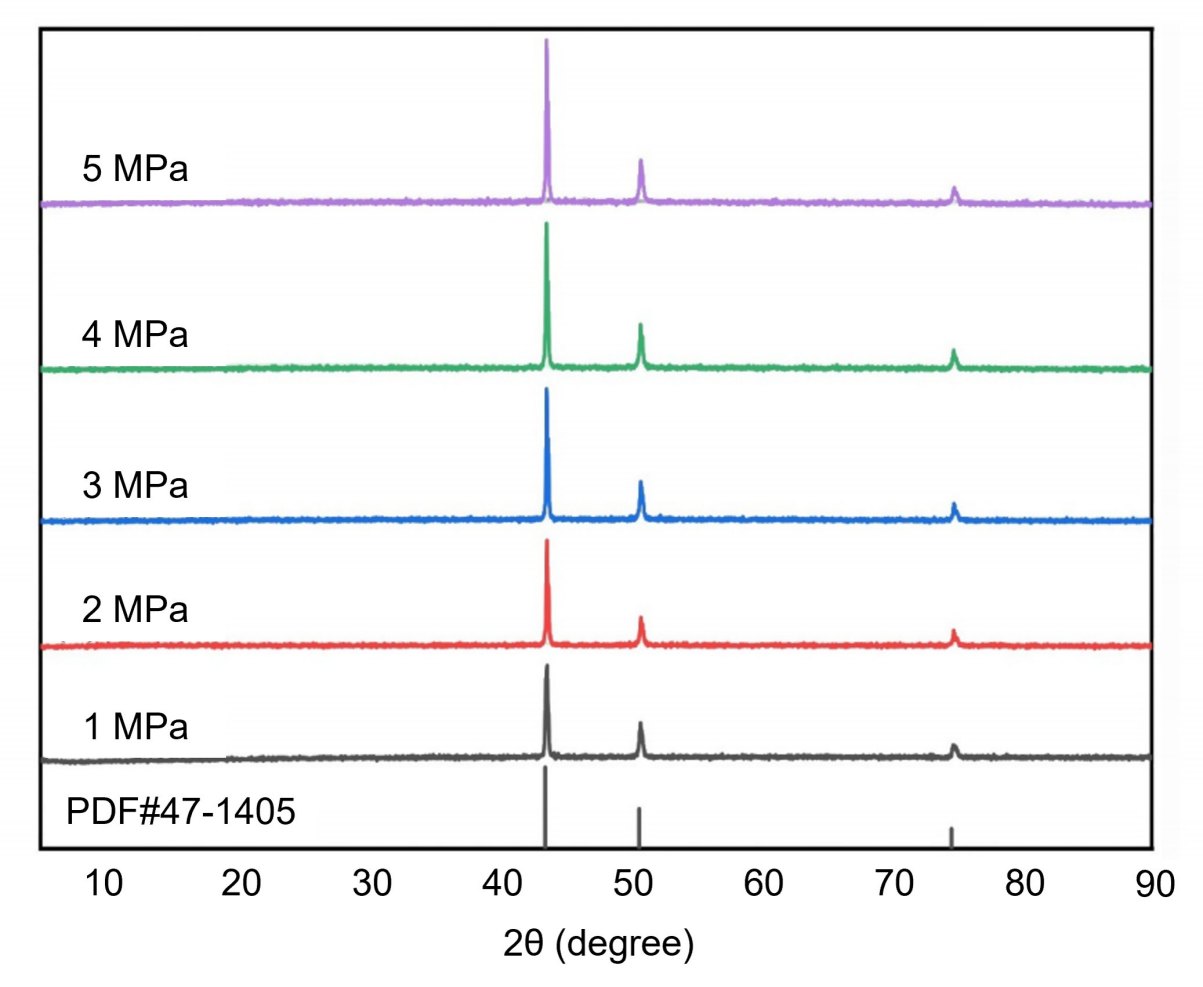
XRD patterns of FeNi50 powder under different pressures.

Figure 9 presents the XRD patterns of FeNi50 powders under different atomization pressures. All diffraction peaks are consistent with the standard PDF card (PDF#47-1405) and correspond to the single face-centered cubic (FCC) γ-(Fe, Ni) solid solution phase. No characteristic peaks of the body-centered cubic (BCC) α-Fe phase are observed, confirming the compositional homogeneity of the alloy powder. The rapid solidification process during gas atomization provides a high cooling rate, which inhibits precipitation of the BCC phase and stabilizes the single FCC structure. The results indicate that the basic crystal structure of the powders remains unchanged under different pressures, suggesting that the atomization pressure primarily affects the morphology and particle size of the powders rather than their phase composition.

#### 3.2.5. Powder Microstructure and Formation Mechanisms

**Figure 10:**
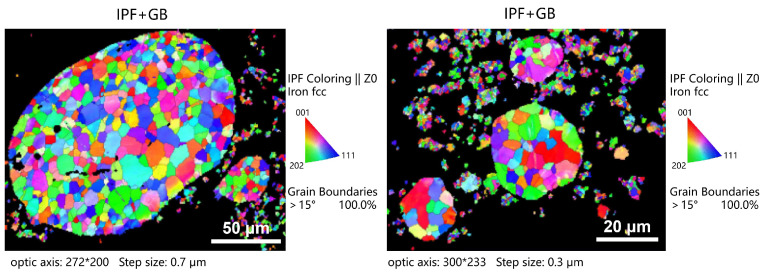
EBSD images of the FeNi50 powder prepared under 5 MPa pressures.

Without heat treatment or etching, grains of varying sizes and clear grain boundaries can be observed on the powder surface. As pressure increases, larger particles still retain distinct grain boundaries, while finer particles exhibit smoother surfaces due to faster cooling rates (Figure 6). EBSD analysis of powder cross-sections under 5 MPa conditions (Figure 10) further reveals that larger particles primarily consist of coarse-grained equiaxed crystals internally. In contrast, fine particles smaller than 15 μm exhibit single-crystal or even amorphous structures internally, a result of extremely high cooling rates completely suppressing grain growth.

The final microstructure morphology of powder (such as equiaxed crystals, dendritic crystals, cellular crystals, or amorphous states) is closely related to its cooling rate. In the highly dynamic and variable process of atomization powder production, directly measuring the cooling rate is extremely challenging. Therefore, it is common to indirectly estimate the cooling rate by evaluating the characteristic dimensions of the microstructure. The functional relationship can be expressed as [28]:

(8) $$D_{g}=b\cdot T^{-n}$$

where $D_{g}$ represents the grain size, lamellar eutectic spacing, or dendrite spacing; *T* represents the cooling rate; and *b* and *n* are constants. The aforementioned microstructural characterization and finite-element simulation results confirm that smaller powder particle sizes correspond to higher cooling rates. In the actual atomization process, higher gas flow velocities (represented by the Mach number) enhance cooling effects, leading to a reduction in the internal grain size of the powder as the Mach number increases [7]. Iron–nickel alloy melt is forcibly cooled, fragmented, and rapidly solidified by high-pressure nitrogen gas in the atomization system. The following section will combine theoretical principles to analyze undercooling, nucleation, and spheroidization behaviors during solidification to explain the causes of unique powder morphologies. During flight within the atomization chamber, metal droplets primarily dissipate heat through convective heat transfer. Given the high flow rate of the atomizing gas ($N_{2}$) and the rapid heat transfer, this study neglects the influence of droplet temperature on the atomizing gas temperature. Based on this, the heat transfer process of metal droplets is governed by the following equation [37]:

(9) $$\begin{array}{cc} C_{pd}\frac{dT_{d}}{dt} & =\Delta H_{d}\frac{df_{s}}{dt}-\frac{6h}{\rho_{d}D}\left(T_{d}-T_{g}\right) \end{array}$$

(10) $$\begin{array}{cc} C_{pd} & =\left(1-f_{s}\right)C_{L}+C_{s}f_{s} \end{array}$$

(11) $$\begin{array}{cc} \Delta H_{d} & =\Delta H_{f}-\left(C_{L}-C_{s}\right)\left(T_{L}-T_{d}\right) \end{array}$$

(12) hc=kg2.0+0.6Re1/2Pr1/3D

(13) $$\begin{array}{cc} \mathrm{Pr} & =\mu_{g}C_{pg}/k_{g} \end{array}$$

where $C_{pd}$ is the specific heat of the solid–liquid two-phase droplet, $C_{L}$ is the specific heat of the liquid droplet, $C_{s}$ is the specific heat of the solid particle, $\Delta H_{d}$ is the comprehensive enthalpy of the droplet, $\Delta H_{f}$ is the melting enthalpy of the metal material, $T_{L}$ is the melting point temperature, $T_{d}$ is the instantaneous temperature of the droplet, $T_{g}$ is the temperature of the atomizing gas, $h_{c}$ is the heat transfer coefficient between the droplet and the atomizing gas, *D* is the diameter of the droplet, $k_{g}$ is the thermal conductivity of the gas, $C_{pg}$ is the specific heat of the gas, $f_{s}$ is the solid phase fraction within the droplet, and Pr (Prandtl number) is the dimensionless Prandtl number. The solidification of metal droplets undergoes four stages: liquid phase cooling, nucleation and recalescence, solidification, and solid phase cooling. The solidification time discussed in this study primarily refers to the duration from the onset of nucleation to the completion of solidification. According to the formula derived by See et al. based on calculating the time required for the release of solidification latent heat, the total time required for the solidification of metal melt is as follows [38]:

(14) $$t_{\mathrm{sol}}=\frac{d\rho_{l}}{6h_{c}}\left[c_{p}\ln f_{0}\left(\frac{T_{l}-T_{\mathrm{gas}}}{T_{m}-T_{\mathrm{gas}}}\right)+\frac{\Delta H}{T_{m}-T_{\mathrm{gas}}}\right]$$

where $\rho_{l}$ is the density of the molten metal, and ΔH is the enthalpy change. Analysis shows that under constant material parameters and gas temperature, the solidification time $t_{\mathrm{sol}}$ is inversely proportional to the heat transfer coefficient $h_{c}$. Combined with Equations (3)–(6) (Reynolds number correlation), an increase in the Reynolds number Re (resulting from higher atomization pressure and gas velocity) will lead to a reduction in $t_{\mathrm{sol}}$. Additionally, a decrease in droplet diameter (due to more thorough secondary fragmentation) will also significantly shorten the solidification time, which aligns with the conclusion of Shi et al. [29] that finer particle sizes correspond to higher cooling rates and shorter solidification times. The spheroidization behavior of droplets is equally crucial. The time required for a metal droplet to form and completely solidify into a spherical particle is referred to as the spheroidization time $t_{\mathrm{sph}}$. This study adopts the classic spheroidization time formula proposed by Nichiporenko:

(15) $$t_{\mathrm{sph}}=\frac{3\pi^{2}\mu }{4V\sigma }\left(R^{4}-r^{4}\right)$$

where $\mu_{l}$ is the dynamic viscosity of the molten metal droplet, *V* is the volume of the droplet, and *R* and *r* are the radii of the droplet before and after spheroidization, respectively. In cyclone atomization, $3\pi^{2}\mu_{l}/4\sigma$ can be regarded as a constant *K*, making it evident that the spheroidization time is proportional to the droplet size. The formation of irregular and near-spherical powder can be reasonably explained by the competitive relationship between spheroidization time and solidification time: when $t_{\mathrm{sol}}<t_{\mathrm{sph}}$, droplets solidify before achieving full spheroidization, leading to the formation of irregular powder. This is more common under high atomization pressures and for fine powder with particle sizes < 50 μm. When $t_{\mathrm{sol}}=t_{\mathrm{sph}}$, spherical powder can theoretically form. When $t_{\mathrm{sol}}>t_{\mathrm{sph}}$, droplets have sufficient time to spheroidize, but this can easily lead to the formation of satellite particles or coarse grains, adversely affecting magnetic properties.

To quantitatively verify the solidification--spheroidization competition mechanism and build explicit correlations between cyclone-atomized powder morphology and theoretical mathematical models, the coupled relationship between spheroidization time and solidification time was systematically analyzed. Key model assumptions, governing formulas, and critical physical parameters were determined (Table 4). Solidification time was calculated via Equations (10)–(14), whereas spheroidization time was derived from Equation (15) with an initial protrusion shape parameter R/r=1.5–1.8 to approximate real near-spherical initial droplets. Based on volume conservation, the spheroidization time was simplified as $t_{\mathrm{sph}}=8.5\;\mu_{l}/\sigma D$. Calculated solidification and spheroidization times for various particle sizes are listed in Table 5. Theoretical calculations demonstrate that solidification time is consistently shorter than spheroidization time for all particle sizes during cyclone atomization. This indicates that molten droplets solidify completely before surface tension drives full spheroidization, resulting in near-spherical rather than perfectly spherical powders, which is well consistent with SEM morphological observations. With increasing particle diameter, the difference between solidification and spheroidization time gradually decreases, meaning larger droplets have sufficient time for spheroidization and a lower probability of irregular morphology, matching experimental statistical results. An inflection point of time-ratio reversal occurs at a critical particle size of 48μm, where prolonged solidification time may induce coarse internal grains. This critical size is highly consistent with the morphological boundary shown in Figure 6 and Figure 7, mathematically validating the solidification--spheroidization competition mechanism for powder morphology regulation.

In actual cyclone atomization processes, powder morphology is mainly regulated by three synergistic factors: First, the competitive relationship between $t_{\mathrm{sol}}$ and $t_{\mathrm{sph}}$ determines the proportion of irregular powder among fine particles. Second, under the combined action of centrifugal and Magnus forces, near-spherical powder tends to form, and the swirling effect avoids direct airflow impact, significantly reducing the likelihood of satellite and hollow particle formation. Third, according to the “non-breakable” theory, particles undergo plastic deformation due to shear and compressive forces during high-speed rotation rather than further fragmentation, resulting in near-spherical powder with lower sphericity.

### 3.3. Analysis of Economy and Manufacturing Cycle

Cyclone atomization exhibits promising industrial application potential for soft magnetic powder production and has been adopted for batch manufacturing in magnetic material enterprises. A systematic economic analysis and manufacturing cost comparison with conventional gas atomization and close-coupled gas atomization are summarized below:

(1) Fine powder yield and raw material saving: As shown in Figure 5C, the −500 mesh fine powder yield of cyclone atomization exceeds 50%, far higher than the value (<10%) of close-coupled gas atomization. To produce 1 ton of −500 mesh ultrafine FeNi50 powder, cyclone atomization requires only 1/5 raw material input of the close-coupled process, which significantly reduces costs of raw materials, gas consumption, electricity, and labor (Table 6, industrial practical data).

(2) Manufacturing cycle shortening: Under the same atomization rate, high fine powder yield shortens powder classification time and waste powder remelting procedure, decreasing the overall production cycle by 40–60%.

(3) Morphology-driven economic gains: High-sphericity, hollow-free, and homogeneous powders improve magnetic core density and magnetic performance, lower resin dosage and power loss, and deliver indirect economic benefits for downstream industries.

(4) Cyclone-atomized FeNi50 soft magnetic powders possess superior magnetic properties, as summarized in Table 7. Magnetic cores were fabricated from −250 mesh powders via identical industrial-standard coating and consolidation processes for fair comparison. Compared with conventional and close-coupled gas atomization counterparts, cyclone gas atomization FeNi50 delivers a higher quality factor, better DC-bias performance, and extremely low core loss. By contrast, FeNi50 powders produced by conventional gas atomization show relatively poor magnetic performance, which originates from its inherent atomization mechanism.

### 3.4. Limitations and Future Perspectives

The cyclone atomization delivers outstanding performance for fabricating high-quality soft magnetic FeNi50 powders, whereas it still has inherent limitations and faces multiple industrialization challenges for practical large-scale engineering application, which are critically discussed as follows:

(1) Narrow stable operational pressure window: The stable working pressure range of cyclone atomization is physically constrained. Low pressure (<1 MPa) results in poor atomization efficiency, while high pressure (>6 MPa) triggers instability of the swirling flow field and shift of shock-wave positions, further causing liquid-column lifting failure and high risks of back-blow nozzle blockage. These pressure-related constraints are highly associated with nozzle geometry and the protrusion length of the guide nozzle. Structural optimization of the spray-disk design, guide-nozzle configuration, and negative-pressure zone layout to elevate the critical back-blow pressure is a core research direction for realizing stable atomization under high-pressure conditions.

(2) Industrial scalability: Process stability has been validated in 250 kg batch pilot tests. For ton-scale industrial upscaling, key challenges remain, including spray-disk array design, symmetrical flow-field control inside the atomization chamber, and development of online replaceable guide-nozzles for rapid process switching. Fortunately, the unique swirling-flow distribution feature of cyclone atomization helps mitigate potential scaling risks, and future research can adopt similarity theory to systematically explore upscaling rules of the atomization process.

(3) Universality for diverse material systems: At present, cyclone atomization has achieved favorable performance only in preparing soft magnetic alloy powders. Its adaptability to pure metal powders and other functional alloy systems requires further experimental verification.

(4) Intelligent process optimization: Future research will focus on intelligent manufacturing strategies. For instance, machine learning algorithms can be combined to precisely screen the stable operational window of critical process parameters such as atomization pressure, and optimize the process boundary of cyclone atomization.

## 4. Conclusions

This work systematically investigates the preparation of FeNi50 powder by cyclone atomization through simulation, experiments, and theoretical analysis. The main conclusions are as follows:

(1) Increasing pressure from 1 to 5 MPa reduces the average particle size ($D_{50}$) from 80.7 μm to 27.9 μm and raises the yield of fine powder (−500 mesh) to 50.0%, significantly higher than that of close-coupled nozzle atomization (<10%).

(2) The relationship of powder morphology evolution with pressure was elucidated. Particles are predominantly near-spherical, with clear grain boundaries at low pressures and blurred boundaries at 5 MPa. Higher pressure increases the fraction of fine particles (<20 μm), which have smooth surfaces, no grain boundaries, and occasional non-spherical shapes without significant agglomeration. Powder morphology is governed by $t_{\mathrm{sol}}$ vs. $t_{\mathrm{sph}}$, aided by centrifugal and Magnus forces in the cyclone field, which promote sphericity while reducing satellites and hollow particles. Some droplets undergo plastic deformation, forming rough near-spherical surfaces.

(3) The formation mechanisms of key defects have been clarified. Irregular powder results from premature solidification; hollow powder from gas entrainment or airflow decay; satellite powder from particle agglomeration driven by velocity gradients. Rough near-spherical powder is typical under swirling flow forces.

(4) Higher pressure refines microstructure via increased cooling rate. Large particles show equiaxed grains, while small particles (<15 μm) form single-crystal or amorphous structures due to rapid solidification.

These findings realize the three core innovations: establishing industrial-scale process--property correlations, revealing the liquid film torsional breakup mechanism via simulation-experiment validation, and quantifying the solidification--spheroidization competitive model for defect regulation. However, this work also reveals that the effective pressure window for cyclone atomization is relatively narrow, as it tends to fail near 6 MPa due to flow field instability. This suggests that future research should focus on optimizing the geometric design of the nozzle (such as the airflow incidence angle and the extension length of the delivery tube) to broaden the operational range for stable atomization. Additionally, while the turbulence model used in this study has been validated for trend prediction, higher-precision models such as large eddy simulation (LES) could be employed in the future to capture the subtle effects of transient fluctuations in the swirling flow field on droplet fragmentation and collisions. Finally, applying the process–structure relationship established in this study to guide subsequent steps—such as insulating coating and compaction molding of the powder, to evaluate the final magnetic core performance—will be a critical next step in advancing the industrialization of this technology.

## Figures and Tables

**Table 1:** Chemical composition of the FeNi50 alloy powder measured by ICP-OES.

Component	C	Si	Mn	P	S	O	Al	Cu	Ni	Fe
Content (wt%)	<0.02	<0.1	<0.01	<0.05	<0.01	<0.1	<0.03	<0.05	48∼51	Remain

**Table 2:** Dimensionless force ratios under typical cyclone-gas-atomization conditions.

Parameter	Expression	Value Range	Dominant Effect
Rep	$\left(\rho_{g}Dv_{p}\right)/\mu_{g}$	1–500	Drag-force regime division
Fr	$\frac{\rho_{l}}{\rho_{g}}\cdot \frac{u_{\theta }^{2}}{u_{r}^{2}}\cdot \frac{L_{c}}{r}$	1–5	Droplet spatial distribution
$\Pi_{M}$	$\frac{3}{4}\cdot \frac{\rho_{g}}{\rho_{l}}\cdot \frac{\omega D}{v_{p}}$	0.2–0.8	Lateral migration of large droplets
$\Phi_{\mathrm{th}}$	$|F_{\mathrm{th}}|/\left(m_{p}a_{p}\right)$	<0.02(D>30μm)–0.3 (D<10μm)	Motion of ultrafine droplets

**Table 3:** Performance comparison between cyclone-GA and conventional atomization technologies for metal powder production.

Process	Sphericity	Satellite	Hollow Fraction	O Content	Apparent Density	Avg. Size
	(%)	Particles	(%)	(ppm)	(g/cm^3^)	(μm)
Conv. GA	92	Many	0.5%	900	4.53	63
Water-A	59	Relatively many	0.1%	3000	3.0	45
Centrifugal-A	98	None	0.2%	200	4.2	78
Cyclone-GA	91	Very few	0.01%	850	5.0	43

Conv. GA: conventional gas atomization; Water-A: water atomization; Centrifugal-A: centrifugal atomization; Cyclone-GA: cyclone gas atomization.

**Table 4:** Key physical parameters for solidification-spheroidization calculation.

Parameter	Symbol	Value	Unit	Basis
Melt density	$\rho_{l}$	7500	$\mathrm{kg}/m^{3}$	Liquid density of Fe-50Ni
Average specific heat	$c_{p}$	600	J/(kg·K)	Liquid-phase average
Latent heat of fusion	ΔH	270	kJ/kg	Typical Fe-Ni alloy value
Initial droplet temperature	$T_{l}$	1923	K	Pouring temp. (1650 °C)
Liquidus temperature	$T_{m}$	1723	K	Approx. 1450 °C
Atomizing gas temperature	$T_{\mathrm{gas}}$	300	K	Room-temp. nitrogen
Gas thermal conductivity	$k_{g}$	0.06	W/(m·K)	High-temp. nitrogen estimation
Gas dynamic viscosity	$\mu_{g}$	$4\times 10^{-5}$	Pa·s	High-temp. nitrogen
Gas density	$\rho_{g}$	1.0	$\mathrm{kg}/m^{3}$	Avg. after atomization expansion
Prandtl number	Pr	0.7	−	Nitrogen
Gas-droplet relative velocity	$v_{\mathrm{rel}}$	See Table 5	m/s	Size-dependent
Surface tension of liquid metal	σ	1.8	N/m	Fe-Ni alloy
Effective viscosity under undercooling	$\mu_{l}$	3.9	Pa·s	Viscosity before recalescence

**Table 5:** Calculated solidification and spheroidization times for powders with different particle sizes.

Diameter	Rel. Vel.	Re	Nu	Heat Transf.	$T_{\mathrm{sol}}$	$T_{\mathrm{sph}}$
D (μm)	$v_{\mathrm{rel}}$ (m/s)			Coeff. $h_{c}$ (W/m^2^K)	(*μ*s)	(*μ*s)
5	15	1.88	2.73	32,760	44	110
10	30	7.50	3.46	20,760	85	220
20	60	30.0	5.05	15,150	230	440
30	90	67.5	6.48	12,960	400	660
40	120	120	7.82	11,730	560	880
48	144	187.5	9.32	11,196	700	1056
60	170	255	10.50	10,500	920	1320
80	200	400	12.65	9488	1400	1760
100	220	550	14.55	8730	1900	2200
120	240	926.5	18.88	7632	2500	2640

Rel. Vel.: relative velocity; Re: Reynolds number; Nu: Nusselt number; Heat Transf. Coeff.: heat transfer coefficient.

**Table 6:** Industrial economic indicator comparison (per ton) of different atomization processes for FeNi50 powder fabrication.

Process	200-Mesh Yield	500-Mesh Yield	Power Cons.	Gas Cons.	Raw Material Cons.	Labor Req.
Conv. GA	40%	5%	12,000 kWh	37.8 t	24.68 t	15 persons
CC-GA	61%	10%	6000 kWh	18.9 t	11.78 t	8 persons
Cyclone-GA	88%	50%	1200 kWh	3.8 t	2.35 t	4 persons

Conv. GA: conventional gas atomization; CC-GA: close-coupled gas atomization; Cyclone-GA: cyclone gas atomization.

**Table 7:** Comparison of magnetic properties of FeNi50 powders prepared by different atomization processes.

Process	Size	*L*	L10A/L0A	L20A/L0A	*Q*	Core Loss @ 50 kHz/100 mT
Conv. GA	−250 mesh	43.4	90%	76.7%	72	487
CC-GA	−250 mesh	41.6	83%	61.0%	38	596
Cyclone-GA	−250 mesh	28.0	96%	85.5%	154	196

*L*: Inductance; *Q*: Quality Factor.

## Data Availability

The original contributions presented in this study are included in the article/Supplementary Material. Further inquiries can be directed to the corresponding authors.

## Supplementary Materials

The following supporting information can be downloaded at: https://www.mdpi.com/article/10.3390/ma19132926/s1, Table S1: Comparison of the average (µd) and variance (σd) of the probability density distribution of FeNi50 powder obtained from experiments and simulations, along with the error.; Tables S2–S6: Particle size sieving results of FeNi50 powder under the different pressure of 1–5 MPa; Table S7: Particle-size parameters (D10, D50, D90 and average diameter) of FeNi50 powder under different atomization pressures from SEM statistics.; Figure S1: SEM-based particle-size frequency distribution of FeNi50 powder under different atomization pressures.; Video S1: Process of the powder production by cyclone atomization.

## Abbreviations

The following abbreviations are used in this manuscript:

CFD	Computational fluid dynamics
VOF	Volume-of-fluid
DPM	Discrete phase model
SMD	Sauter mean diameter
XRD	X-ray diffraction
SEM	Scanning electron microscopy
