# Supplementary material for: Simultaneous Efficient Fragmentation and Spheroidization: Cyclone Atomization Enables Defect-Free, High-Yield FeNi50 Powder
Source: Materials (Basel). 2026 Jul 7;19(13):2926. doi: 10.3390/ma19132926 (PMC13363360; doi:10.3390/ma19132926)
Supplement: Supplementary file 1 [file materials-19-02926-s001.zip › Figures and Tables.pdf]

Article

Simultaneous Efficient Fragmentation and Spheroidization: Cyclone Atomization Enables Defect-Free, High-Yield FeNi50 Powder

Kai Kang [1,*] Shasha Huang [2,*] ,Kuanguang Hu [3], Qiang Han [3] and Deliang Zhang [1]

[1] School of Materials Science and Engineering, Northeastern University, Shenyang, 110819, China

[2] School of Chemistry and Chemical Engineering, Linyi University, Linyi, 276000, China

[3] Qingdao Ruinuotu Intelligent Technology Co., Ltd., Qingdao, 266109, China

* Correspondence: kangkai_2025@126.com (K.K.); huangshasha@lyu.edu.cn (S.H.)

Table S1. Comparison of the average (μ_d) and variance (σ_d) of the probability density distribution of FeNi50 powder obtained from experiments and simulations, along with the error.

Pressure (MPa)		Experiment (m)	Simulation (m)	Error (%)
1	μ_d	80.75 ± 1.01	82.365 ± 1.21	2.11
	σ_d	58.56 ± 1.82	62.80 ± 2.35	7.24
2	μ_d	43.61 ± 2.55	45.14 ± 2.61	3.52
	σ_d	32.07 ± 0.94	33.41 ± 2.55	4.17
3	μ_d	43.52 ± 0.44	45.48 ± 1.65	4.53
	σ_d	31.67 ± 0.84	32.65 ± 1.98	3.15
4	μ_d	39.02 ± 0.68	40.97 ± 2.88	5.04
	σ_d	26.23 ± 0.55	28.48 ± 0.99	8.81
5	μ_d	27.98 ± 1.34	29.66 ± 1.77	6.05
	σ_d	17.49 ± 0.43	18.21 ± 0.85	9.23

Table S2. Particle size sieving results of FeNi50 powder under the pressure of 1 MPa.

Mesh	Particle size (m)	Cumulative (%)	Mesh	Particle size (m)	Cumulative (%)
20	850	100.00	270	53	35.00
25	710	100.00	325	45	30.15
30	600	100.00	400	38	25.90
35	500	100.00	450	32	22.13
40	425	100.00	500	28	19.41
45	355	100.00	600	23	15.71
50	300	100.00	700	20	13.33
60	250	100.00	800	18	11.71
70	212	99.23	1000	13	7.39
80	180	94.67	1250	10	4.54
100	150	84.16	1670	8.5	3.21
120	125	72.04	2000	6.5	1.84
140	106	62.18	5000	2.5	0.00
170	90	54.38	8000	1.5	0.00
200	75	47.17	10000	1.3	0.00
230	63	40.85	12000	1	0.00

Table S3. Particle size sieving results of FeNi50 powder under the pressure of 2 MPa.

Mesh	Particle size (m)	Cumulative (%)	Mesh	Particle size (m)	Cumulative (%)
20	850	100.00	270	53	57.81
25	710	100.00	325	45	51.19
30	600	100.00	400	38	44.76
35	500	100.00	450	32	38.48
40	425	100.00	500	28	33.92
45	355	100.00	600	23	27.80
50	300	100.00	700	20	23.89
60	250	100.00	800	18	21.18
70	212	100.00	1000	13	13.85
80	180	99.90	1250	10	9.04
100	150	98.77	1670	8.5	6.68
120	125	95.36	2000	6.5	3.85
140	106	89.62	5000	2.5	0.06
170	90	82.40	8000	1.5	0.00
200	75	73.50	10000	1.3	0.00
230	63	65.32	12000	1	0.00

Table S4. Particle size sieving results of FeNi50 powder under the pressure of 3 MPa.

Mesh	Particle size (m)	Cumulative (%)	Mesh	Particle size (m)	Cumulative (%)
20	850	100.00	270	53	56.60
25	710	100.00	325	45	51.06
30	600	100.00	400	38	45.67
35	500	100.00	450	32	40.39
40	425	100.00	500	28	36.47
45	355	100.00	600	23	30.96
50	300	99.98	700	20	27.21
60	250	99.70	800	18	24.49
70	212	98.94	1000	13	16.64
80	180	97.53	1250	10	11.16
100	150	94.52	1670	8.5	8.38
120	125	89.76	2000	6.5	4.95
140	106	83.80	5000	2.5	0.20
170	90	77.19	8000	1.5	0.00
200	75	69.63	10000	1.3	0.00
230	63	62.85	12000	1	0.00

Table S5. Particle size sieving results of FeNi50 powder under the pressure of 4 MPa.

Mesh	Particle size (m)	Cumulative (%)	Mesh	Particle size (m)	Cumulative (%)
20	850	100.00	270	53	61.27
25	710	100.00	325	45	55.13
30	600	100.00	400	38	49.07
35	500	100.00	450	32	43.02
40	425	100.00	500	28	38.48
45	355	100.00	600	23	32.10
50	300	100.00	700	20	27.83
60	250	99.98	800	18	24.79
70	212	99.74	1000	13	16.32
80	180	98.79	1250	10	10.64
100	150	96.38	1670	8.5	7.85
120	125	92.67	2000	6.5	4.55
140	106	87.91	5000	2.5	0.19
170	90	82.28	8000	1.5	0.00
200	75	75.11	10000	1.3	0.00
230	63	68.09	12000	1	0.00

Table S6. Particle size sieving results of FeNi50 powder under the pressure of 5 MPa.

Mesh	Particle size (m)	Cumulative (%)	Mesh	Particle size (m)	Cumulative (%)
20	850	100.00	270	53	76.03
25	710	100.00	325	45	69.41
30	600	100.00	400	38	62.59
35	500	100.00	450	32	55.49
40	425	100.00	500	28	50.02
45	355	100.00	600	23	42.14
50	300	100.00	700	20	36.74
60	250	100.00	800	18	32.88
70	212	100.00	1000	13	22.05
80	180	100.00	1250	10	14.76
100	150	99.79	1670	8.5	11.14
120	125	98.83	2000	6.5	6.74
140	106	96.83	5000	2.5	0.51
170	90	93.78	8000	1.5	0.00
200	75	88.76	10000	1.3	0.00
230	63	82.77	12000	1	0.00

Table S7. Particle-size parameters (D10, D50, D90 and average diameter) of FeNi50 powder under different atomization pressures from SEM statistics.

Pressure / MPa	D10 / μm	D50 / μm	D90 / μm	Average diameter / μm
1	30.93 ± 2.82	59.73 ± 3.57	93.82 ± 4.91	58.26 ± 19.63
2	22.84 ± 2.17	40.80 ± 2.94	63.98 ± 3.86	42.05 ± 14.61
3	19.89 ± 1.93	36.54 ± 2.48	49.61 ± 3.22	36.23 ± 12.43
4	12.33 ± 1.45	32.72 ± 2.09	43.11 ± 2.87	30.89 ± 11.07
5	8.29 ± 1.02	23.91 ± 1.64	35.16 ± 2.11	22.58 ± 6.69

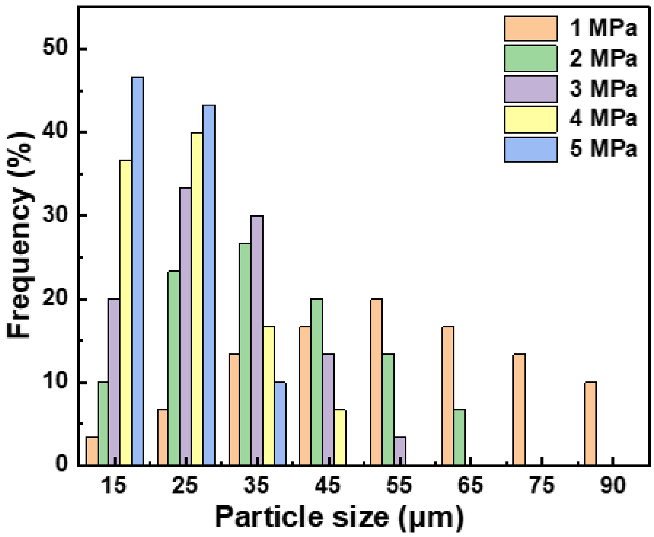

Figure S1. SEM-based particle-size frequency distribution of FeNi50 powder under different atomiza-tion pressures.
